# Notes and News

**Published:** 1897-03-27

**Authors:** 


### NOTES AND NEWS.

(Collected and Communicated.)

HOSPITAL MEETINGS.

London Homœopathic Hospital, Great Ormond Street.—Annual general meeting, Friday, March 26th, 3 p.m., in board-room of the New Hospital, Great Ormond Street.

Royal London Ophthalmic Hospital, Moorfields.—Annual meeting in board room of Hospital, Tuesday, March 30th, 12 o'clock noon.

The St. John's Ambulance fête to commemorate the Diamond Jubilee will be held at the Crystal Palace on May 6th.

Both the Allan and Dominion Lines to Canada have reduced their fares for members of the British Medical Association visiting Montreal for the meeting, providing they sail before August 5th.

A medical skiagraphical society is being formed, which, if conducted on liberal lines, it should prove very useful. It should assist in perfecting the technique of the art, and afford means of comparison amongst experimenters who without this would spend much unnecessary time in testing unprolific ground.

Both Dr. Yersin and Dr. Haffkine appear to be meeting with great success in the use of the serum respectively advocated by them. The serum used by Dr. Yersin is more especially directed to the cure, while that used by Dr. Haffkine aims at the prevention of the plague. The latest reports received are to the effect that Dr. Yersin's results, in some very bad cases, have been so good as to be surprising even to himself.

Sir Henry Littlejohn, who has succeeded Sir Douglas Maclagan as professor of forensic medicine at Edinburgh, took his medical degree at the same university. He is a Fellow of the Royal College of Surgeons of Edinburgh, and has acted as its president; also of the Edinburgh Medico-Chirurgical Society, and as medical officer of health for the City. His services in the direction of hygiene and sanitation have been invaluable. He has always been a successful and popular lecturer, and his candidature to the chair of forensic medicine received enthusiastic support both locally and from many parts of the country.

Mr. Frederick Treves, F.R.C.S., has been appointed Surgeon-in-Ordinary, and Mr. Alan Reeve Manby, M.D., Surgeon-Apothecary to H.R.H. the Duke of York, at Sandringham.

A new special Hospital for Diseases of the Stomach is about to be opened at Philadelphia. Connected with it are to be research laboratories and a department concerned with the adulteration of food.

A public meeting will be held in aid of the National Jenner Memorial in the theatre of the University of London, Burlington Gardens, on Wednesday, March 31st, at four p.m. The Duke of Westminster will preside.

Although it appears that it is contemplated to establish a central military hospital for London, it is not likely that it will be ready and occupied for some time to come. The site mentioned is that previously occupied by the old Milbank prison.

The Royal Sea Bathing Infirmary at Margate is issuing an appeal for £2,000. Certain defects in the building require immediate attention, and for this purpose the mentioned sum is required. The infirmary is not nearly so liberally supported as it should be.

The medical staff of St. George's Hospital assembled a large number of interested friends at a soirée last week to view the new operating theatres and laboratories of the hospital, which are now in use. The theatres have been rendered as complete in every detail as possible, and especial attention has been given to the ventilation.

According to latest reports from Bombay, there is still determined opposition in the native town to the measures taken for the segregation of plague cases. In Karachi, however, such opposition has almost entirely ended, and great energy has been shown in battling with the plague. Throughout Sind medical inspection of travellers is frequent, but it is a matter of great difficulty to prevent their unrestricted movement, and as a result of the concealment of cases plague is now established in Haiderabad and Sukkur. The Plague Committee in the various centres, however, are doing their utmost in the way of compulsory isolation of sick and the segregation of those probably infected.

The board of management of the Royal Asylum at Montrose have lately performed a duty which must have been one of mingled pleasure and pain. Dr. Howden, who had held the post of medical superintendent of the asylum for upwards of a generation, sent in his resignation. It must have been painful indeed for the board to accept this resignation from one who had so long been their trusted and valued servant; but there was also an element of pleasure, for the members were able to vote as pension an annual sum equal to two-thirds of the salary lately received by Dr. Howden. This is the highest pension permissible by the Lunacy Acts, and the board did not consider it sufficient to meet the merits of the case. It was therefore decided to appoint Dr. Howden consulting physician to the asylum at a salary of £100 a year. The duties of this office can hardly be more than nominal, and it was a graceful action on the board to think of it and pass it. Would that such instances were more common! Dr. Howden's successor is to begin at £700 a year, presumably with the usual emoluments.

